# Nitrogen/sulfur dual-doped micro-mesoporous hierarchical porous carbon as host for Li-S batteries

**DOI:** 10.3389/fbioe.2022.997622

**Published:** 2022-09-26

**Authors:** Liping Zhao, Lihe Zhao, Ye Zhao, Gang Liu

**Affiliations:** ^1^ Institute of Chemical and Industrial Bioengineering, Jilin Engineering Normal University, Changchun, China; ^2^ Daqing Oilfield Design Institute Co, Ltd, Daqing, China; ^3^ FAW Tooling Die Manufacturing Co, Ltd, Changchun, China

**Keywords:** Li-S batteries, nitrogen/sulfur dual-doped, porous carbon, micro-mesoporous, hierarchical structure

## Abstract

A simple hydrothermal process employing sucrose and glutathione as the source of carbon and nitrogen-sulfur, respectively, a porous carbon/sulfur composite material doped with nitrogen and sulfur (NSPCS) was synthesized. The detailed structure information of the material was characterized by X-ray diffraction (XRD), X-ray photoelectron spectroscopy (XPS) and Raman spectroscopy. The morphology information was investigated through Scanning Electron Microscope (SEM) methods. Structure of the pores and pore size distribution were investigated employing N_2_ adsorption-desorption isotherm. The material was treated Thermogravimetric analysis (TGA) in order to know the weight ratio of sulfur. The synthesized NSPCS composite produced high specific capacity, excellent rate performance and exceptionally good cycle stability when used as the positive electrode in Li-S batteries.

## Introduction

Because of high theoretical specific energy (2,600 Wh kg^−1^), non-toxicity and abundant natural reserves, Li-S batteries are expected to become the next generation of new energy storage systems (Chen et al., 2022; Wang et al., 2022). Lots of interesting results on Li-S batteries have been reported (Kim et al., 2020; Ahn et al., 2022; Qian et al., 2022; Wei et al., 2022; Yu et al., 2022; Zhao et al., 2022). However, Li-S batteries still face two important challenges. One is the poor electrical conductivity of the elemental sulfur and discharge products, the other is the dissolution of polysulfide compounds during charge-discharge process causes rapid capacity loss, which limits its practical application (Evers and Nazar, 2012; Yang et al., 2013). So, in order to solve the problems of low electron and ion conductance of sulfur, compounding with carbon to prepare sulfur/carbon composites is widely used (Jin et al., 2022; Xu et al., 2022; Yu et al., 2022). Porous carbon are broadly applied to enhance the capability of Li-S batteries by adjusting pore structure, surface chemistry and other properties. Extensive literature reports have indicated that the polysulfide dissolution can be inhibited by increasing the contact between sulfur and conductive matrix by employing porous carbon materials having large surface area (Luna-Lama et al., 2022; Ma et al., 2022). Depending on the size of the pores, there can be three types of porous carbon namely, macroporous, microporous and mesoporous with each having different effect on the Li-S batteries. Due to open structure with large pore size, the shuttle effect of polysulfides cannot be prevented by carbon having macroporous structure. In contrary, enhanced surface area and decreased pore diameter result in good dispersibility of sulfur, resulting in increased contact with the conductive matrix for microporous carbon (Liang et al., 2009; Wang et al., 2021). The increased specific surface area also improves the physical adsorption behavior of polysulfide compounds in microporous carbon, greatly delaying the decomposition of polysulfide compounds in the electrolytic solution (Hong et al., 2018; Qian et al., 2019). Nevertheless, low sulfur loading reduces the overall energy density of Li-S batteries, which is a result of the carbon materials with microporosity having significantly smaller pore volumes. In comparison to the microporous carbon, the distribution of pore diameter for mesoporous carbon is larger, which accommodates more sulfur to increase the loading of sulfur (Liu et al., 2021a; Yang et al., 2021a). But, presence of excessive sulfur inside mesoporous carbon prevents the formation of good electrical contact between sulfur and the pore walls of conductive carbon. This prevents the full utilization of sulfur leading to reduced rate of utilization. In addition, the larger pore diameters of the mesopores accelerate the dissolution of polysulfides, resulting in the loss of sulfur-active compounds and a weakened cycle stability (Zhao et al., 2019). Hence, taking advantage of both the types of micropores and mesopores, hierarchical porous carbon can be designed consisting of both the pores to increase the Li-S batteries capability. Lots of studies have already shown that the micropores can provide space for storing sulfur, while the mesopores can store polysulfides dissolved in the electrolyte. Such micro-mesoporous hierarchical pore structure is more conducive to loading a large amount of sulfur while better limiting the dissolution of polysulfides, thereby improving the Coulombic efficiency and cycle stability (Yang et al., 2021b; Qiu et al., 2021).

Besides, more and more studies have also reported that doping of heteroatoms [such as nitrogen (Cheng et al., 2022; Lin et al., 2022), phosphorus (Liu et al., 2021b; Huang et al., 2021), sulfur (Li et al., 2018; Zhang et al., 2020), boron (Yang et al., 2014; Eroglu et al., 2020) etc.,] in carbon materials can result in the enhancement of conductivity and electrochemical activity of the material. It has been proven that nitrogen-doped carbon materials can improve the interaction between sulfur and the carbon matrix, assist in inhibiting the shuttle effect of polysulfides, and then enhance the interaction between nitrogen doping site functional groups and polysulfides (Jing et al., 2022). Various research reports have indicated improved cycle performance for sulfur-doped carbon materials as a result of enhanced affinity of polysulfide’s towards the carbon matrix (Yang et al., 2016). Additionally, calculations based on the density functional theory, diffusion experiments, and XPS analysis have all produced strong evidence for a considerable interaction between N and Li_2_Sn atoms in the carbon matrix. Simultaneously, incorporating heteroatoms like S and N, the conductivity of the carbon support can be enhanced which results in enhanced adsorption of S on the carbon based sustenance material with reduced dissolution of lithium polysulfide which results in improved sulfur utilization rate (Tan et al., 2020; Lu et al., 2021). As a result, greater attempts have been made to utilize dual-doped carbon matrix materials that include sulfur and nitrogen for increasing the performance of Li-S batteries (Ding et al., 2019; Lu et al., 2021).

Based on the above analysis, we attempt to design a new type of carbonaceous material, which not only has a high specific surface area and a hierarchical pore structure, but also has a special surface chemistry to improve the interaction between polysulfide and carbon matrix. However, in most cases, structure adjustment and functionalization are two separate processes, which leads to a more complicated manufacturing process (Shi et al., 2019). Furthermore, functionalization after the formation of a complex pore structure may result in uneven doping and a low doping level (Duan et al., 2019). In this work, we proposed a simple and effective method to obtain a N, S dual-doped porous carbon microspheres (NSPC) with a hierarchical pore structure. During the preparation process, we introduced sucrose as the carbon source and glutathione as the dual function agent, through a simple hydrothermal method and subsequent KOH activation. As far as we know, glutathione is a tripeptide including γ-amide bond and sulfhydryl group. Glutamic acid, cysteine and glycine are its constituents. Due to the inclusion of amino (-N_2_H) and sulfhydryl (-SH) groups, it is a rich source of both sulfur and nitrogen. Glutathione serves as a functional agent by supplying sulfur and nitrogen, but it also regulates pore structure, resulting in the presence of micropores and mesopores in the produced carbon sphere with hierarchical porous structure. The results indicates that the N, S dual-doped porous carbon microspheres (NSPC) own high specific surface area, large pore volume and wide pore size distribution, which can load enough sulfur and can tolerate the volume expansion of sulfur during charge-discharge process. Such structure can provide fast ion and electron transport channels as well. Due to the synergistic effect of pore structure and surface functionalization, the N, S dual-doped porous carbon microspheres combined with sulfur composite (NSPCS) as positive electrode material for Li-S batteries exhibits high specific capacity, exceptionally good cycle stability and excellent rate performance. It should be emphasized that present work was able to introduce two heteroatoms like N and S simultaneously by employing only one reagent, glutathione. Moreover, the source of carbon, sucrose, can be obtained easily, cheaper, and easy to handle. Thus the whole synthesis process is low cost and simple. And experimental results prove the co-existence of mesopores and micropores in the hierarchical pore structure of the synthesized NSPCS material which showed relatively superior electrochemical performance.

## Experimental

### Synthesis of samples

Firstly, 6 g sucrose (obtained from Tianjin Damao Chemical Reagent Factory) was dissolved in 70 ml deionized water to become an evenly solution. With stirring, 1.2 g glutathione (obtained from Tianjin Damao Chemical Reagent Factory) was added into the solution. Then the mixture was transferred into a hydrothermal reactor and then treated at 200°C for 12 h. After hydrothermal process, the product was collected by centrifugation treatment. Next, washed the product with absolute ethanol and deionized water several times, and then dried in an oven at 80°C overnight. Hereafter, mixed the sample with KOH (1:3 in mass ratio) uniformly and heat-treated in a tube furnace at 800°C for 2 h under N_2_ atmosphere. After washing with 1 M HCl and deionized water repeatedly, dried at 80°C in air over night, the N, S double-doped porous carbon (NSPC) was synthesized. Hereafter, the NSPC and the sublimed sulfur were solid-phase mixed in a mass ratio of 1:3, and the grinding time should be more than 30 min to mix evenly, and an appropriate amount of carbon disulfide was added during the grinding process to increase the fluidity of the sulfur. Then, the ground samples were placed in a vacuum oven at 155°C for thermal diffusion, and the temperature was set at 155°C because elemental sulfur had the best fluidity at this temperature. The corresponding sulfur-containing composite materials can be obtained by grinding the samples after thermal diffusion. Finally, the N, S double-doped porous carbon/S (NSPCS) composites was obtained. The carbon material without adding glutathione (labeled as PCS) were also obtained.

### Characterizations of samples

The physical properties of the samples were characterized by XRD, Raman, XPS, SEM, BET, and TGA methods, in order to obtain the structure, composition, morphology, specific surface area, pore structure, and sulfur loading information of the samples.

X-ray diffraction (XRD) instrument used an X'Pert PRO X-ray diffractometer produced in the Netherlands. The test conditions are: the radiation source is Cu-Kα, the wavelength is 1.5406 Å, the scanning speed is 10°/min, the tube voltage is 40 kV, the tube current is 80 mA, the scanning step size is 0.02°, and the scanning angle range is 10–80°. X-ray Photoelectron Spectroscopy (XPS) instrument model is Axis Ultra DLD (Kratos). The light source used in the test is Al-Kα X-ray, and the background vacuum is 3 × 10^−7^ Pa. Raman spectroscopy (Raman) instrument used Renishawln Via-plus analyzer from Renishaw Company, United Kingdom, and the laser wavelength is 633 nm. Scanning Electron Microscope (SEM) instrument used Hitachi S-4700 produced in Japan, and the acceleration voltage is 25 kV. The nitrogen adsorption and desorption test was used to analyze the specific surface area and pore size distribution of the samples. The instrument used Micromeritics ASAP 2010 and Micromeritics ASAP 2020 produced in the United States. Thermogravimetric analysis (TGA) was carried out by using a thermogravimetric analyzer of the model SDT Q600 produced by TE Company in the United States. The atmosphere used in the test is nitrogen, the test temperature range is from room temperature to 600°C, and the heating rate is 10°C/min.

### Producing of electrodes and batteries assembly

The prepared active materials, conductive agent and binder are weighed according to the mass ratio of 7:2:1, and then an appropriate amount of N-methylpyrrolidone (NMP) was added to prepare a black with appropriate viscosity slurry. The binder selected in the experiment was a mixture of polyvinylidene fluoride (PVDF) and NMP at a mass ratio of 1:10, and the dissolution temperature was 50°C. The conductive agent selected in the experiment was acetylene black (Super-P). The prepared black slurry can be coated on a clean aluminum sheet after 2 h of magnetic stirring. The coated aluminum sheet was dried at 60°C for 6 h and then put into a tableting machine for tableting at a pressure of 18 MPa. After final drying of 6 h, the obtained positive electrode was cut into a circle (diameter of 13 mm). Li metal was used as the negative electrode. The obtained pole piece was further stored in an argon filled glove box prior to use. The surface active material on the lithium-sulfur battery’s positive sheet has a sulfur loading of 2 ± 0.3 mg·cm^−2^. The battery’s precise capacity was computed taking into account the overall amount of active materials.

The electrolyte was prepared in a glove box. The electrolyte is a solution of 1 M LiTFSI (Shanghai Macleans Biochemical Technology Co., Ltd.) in DOL/DME (Shanghai Macleans Biochemical Technology Co., Ltd.) solution (volume ratio 1:1), and contains 1% LiNO_3_ additives (Shanghai Macleans Biochemical Technology Co., Ltd.). Stirring for a long enough time until a homogeneous solution is obtained. Each coil cell had 0.05 ml of the electrolyte. All the test batteries contained CR2032 coin cells, with celgard 2,400 being used as the separator. A dry glove box (Vigor Technology Co., Ltd.) with 0.3 ppm being the maximum content of H_2_O and O_2_ and filled with high purity Ar gas was employed to assemble all the batteries. After standing for a period of time, electrochemical performance testing was performed.

### Electrochemical measurements

The battery assembled by Shanghai Chenhua electrochemical workstation (chi660D) was used for Cyclic voltammetry (CV) test. The scanning rate is 0.1 mV·s^−1^ and the voltage range is 1.5–3 V.

After the assembled batteries were put aside for 3 h, the land CT2001A multi-channel battery detection system produced by Wuhan LANHE Electronics Co., Ltd. was used to test the constant current charge and discharge of the batteries. The voltage range of the test is 1.5–3 V, the current density of the test ranges from 0.1 C to 2 C as required, and the test condition is room temperature.

Shanghai Chenhua electrochemical workstation (chi660D) is used to test the electrochemical impedance of the assembled battery to obtain the internal impedance data of the corresponding battery. The frequency range of the test is 0.01 Hz–100 kHz, the voltage amplitude of the test is 5 mV, and the test condition is room temperature.

## Results and discussion

### Characterization of samples

**FIGURE 1:**
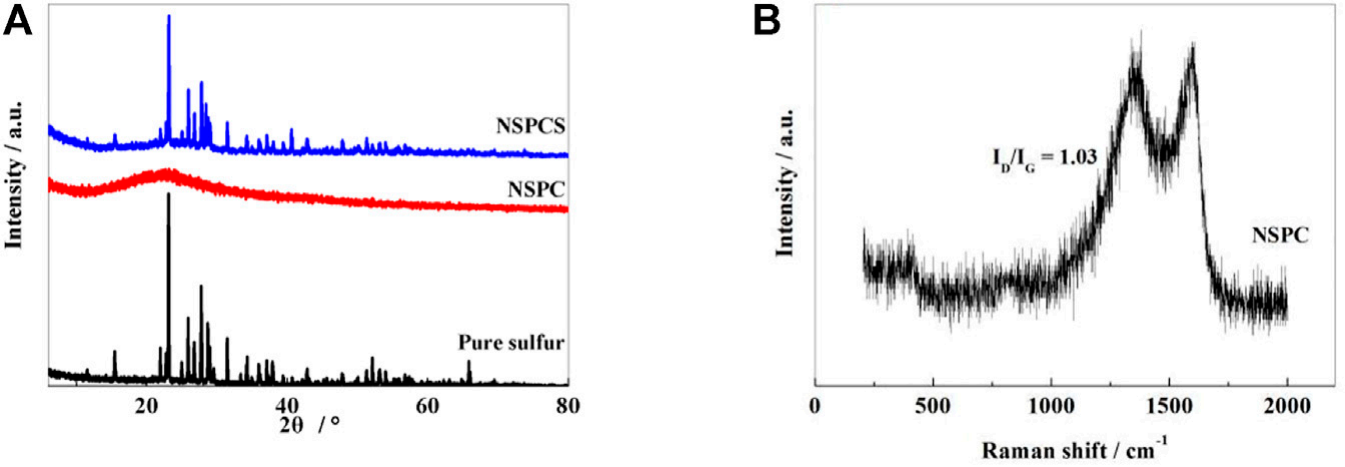
XRD patterns of pure sulfur, NSPC and NSPCS samples **(A)**, Raman spectra of NSPC samples **(B)**.

XRD spectrum (Figure 1A) shows a distinct wide and weak peak near 23.2° for the NSPC composite corresponding to (002) plane of graphite and thus denoting the presence of amorphous carbon in NSPC. Subsequent to thermal combination with sulfur, an obvious diffraction peak corresponding to sulfur can be seen in NSPCS together with a characteristic carbon peak at 23.2°, thus indicating well integration of sulfur into the NSPC matrix, and sulfur in the outer space of the carbon spheres may be responsible for the sulfur diffraction peak.

From the Raman spectrum of NSPC shown in Figure 1B, two characteristic peaks corresponding to the D band and G band of the carbon material can be observed near 1,340 and 1,596 cm^−1^, respectively. The vibration of graphene structure is responsible for the characteristic G band while the vibration corresponding to the carbon present outside the graphite structure is responsible for the D band. The intensity ratio of the two peaks (I_D_/I_G_) infers the degree of disorder and the conductivity of carbon matrix (Yang et al., 2005). After calculating, the ratio of I_D_/I_G_ of NSPC is 1.03, which indicates that the material has relatively good conductivity.

**FIGURE 2:**
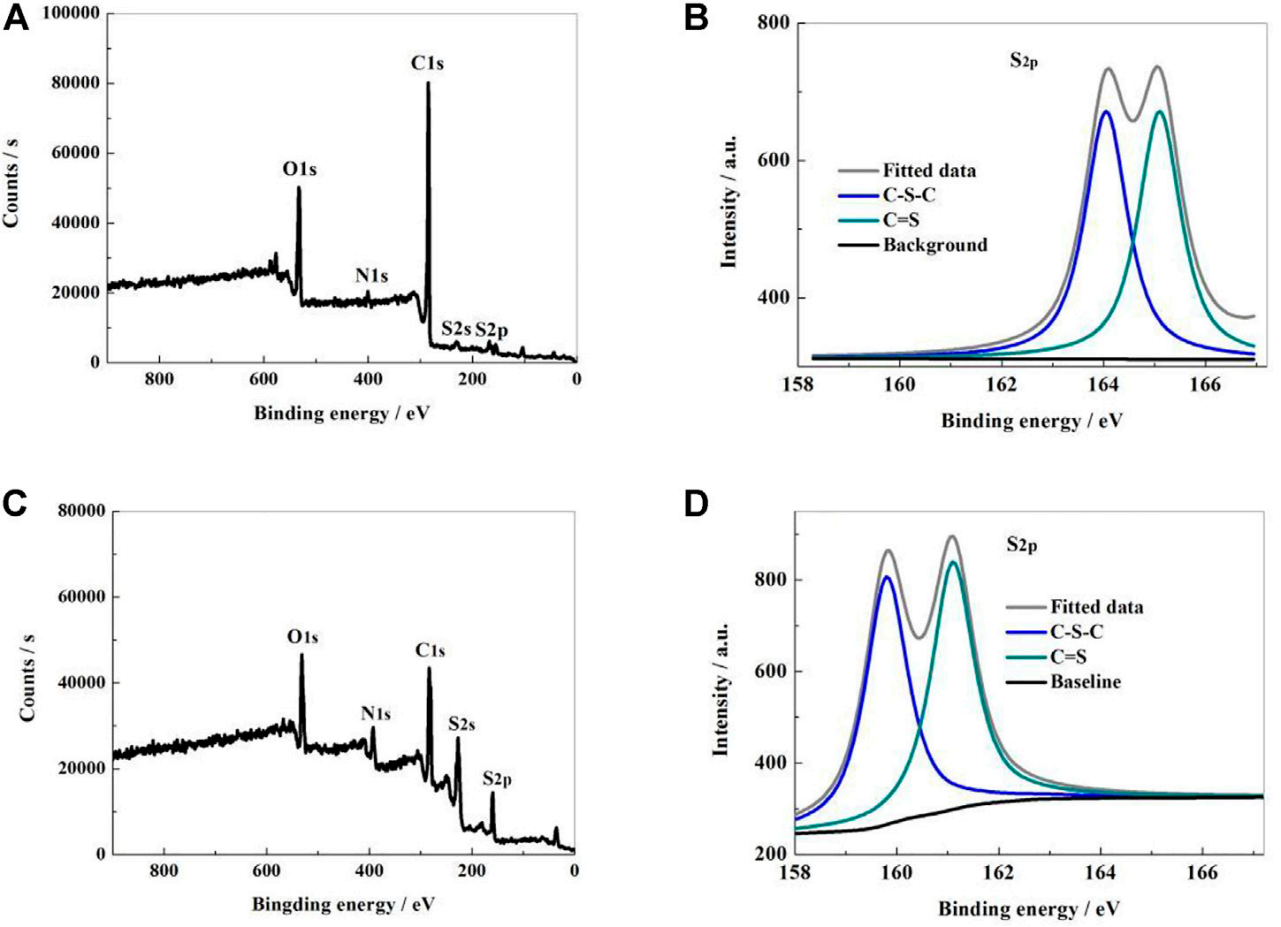
XPS survey of NSPCS **(A)** and NSPC **(C)**; high resolution spectra of S2p in NSPCS **(B)** and NSPC **(D)**.

The microstructure, element content, electron binding energy and bonding state of the sample surface were analyzed by X-ray photoelectron spectrometer (XPS) method. From Figure 2A, it can be shown that the XPS survey spectrum of NSPCS contained five apparent characteristic peaks located at 532, 400, 285, 228, and 168 eV, which corresponds to O1s, N1s, C1s, S2s, and S2p characteristic peaks. From the high resolution XPS spectral analysis of S2p (Figure 2B), two characteristic peaks near 164.05 and 165.1 eV can be seen corresponding to C-S-C and C=S bonds, respectively. In compared to the NSPC sample, the NSPCS demonstrated a greater contact between the sulfur and carbon substrate after combining with the substrate (Figures 2C,D). This collaboration successfully improves the electrochemical performance by limiting the impact on polysulfides and sulfur during the discharging and charging process.

**FIGURE 3:**
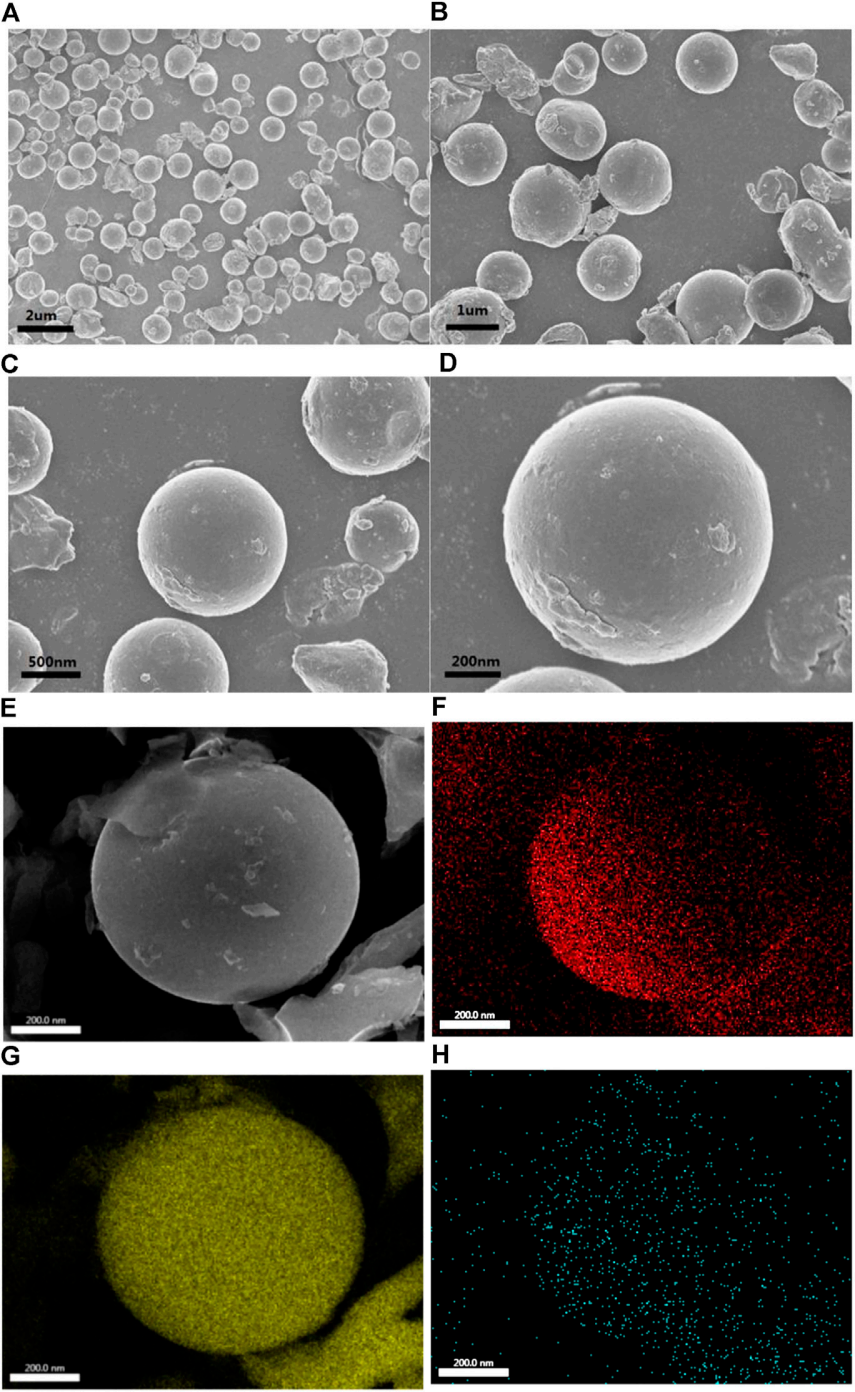
SEM results of NSPCS **(A–E)** and corresponding elemental mappings of C **(F)**, S **(G)**, N **(H)**.

Scanning electron microscope (Figures 3A−E) and elemental surface scan (Figures 3F−H) were carried out on the NSPCS composite material for exploring the morphology and structure of the composite further. From the SEM pictures, the NSPCS composite material shows a spherical structure which is uniform and has a diameter of about 1 µm. Additionally, not many sulfur particles were observed outside the carbon ball which indicates significant penetration of sulfur into the carbon matrix. Even distribution of sulfur and nitrogen atoms in the porous carbon sphere matrix is observed from the elemental analysis of the NSPCS (Figure 3F−H).

**FIGURE 4:**
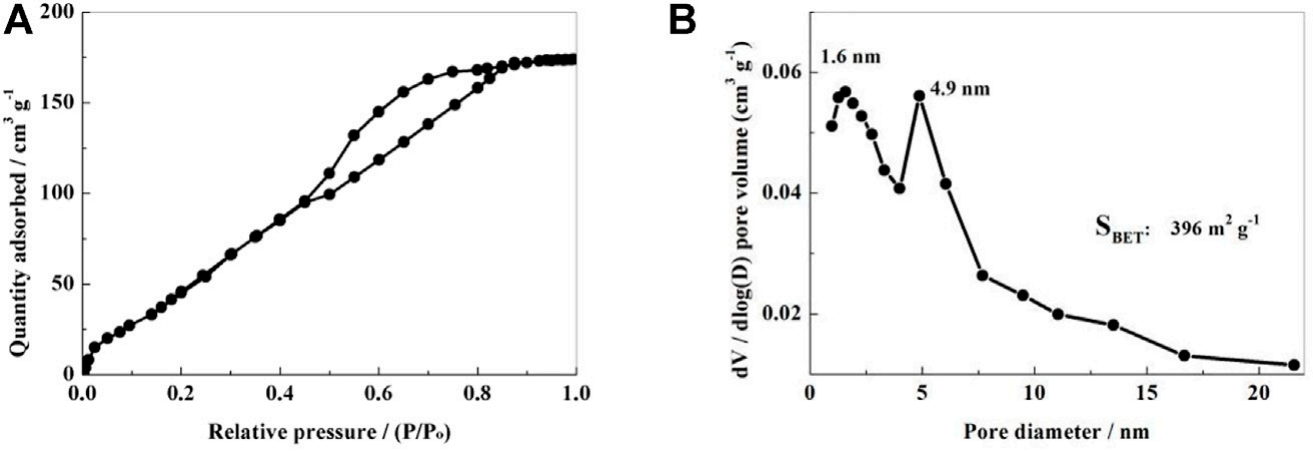
N_2_ adsorption-desorption isotherms **(A)** and the pore size distribution **(B)** of NSPC.

The NSPC’s specific surface area and pore structure were determined using the N_2_ adsorption-desorption isotherms. The equilibrium isotherm of NSPC sample can be found to be a mixture of IUPAC I and IV type curves as seen in Figure 4A, indicating wide distribution of the pore diameter of the NSPC composite materials from micropores to mesopores. Corresponding pore size distribution (Figure 4B) shows two narrow peaks with measurement near 1.6 and 4.9 nm for NSPC. This mixed structure with micropores and mesopores should be able to prevent polysulfide dissolution and enhance the efficient mobility of lithium ions. The NSPC sample’s specific surface area was determined to be 396 m2·g^−1^.

**FIGURE 5:**
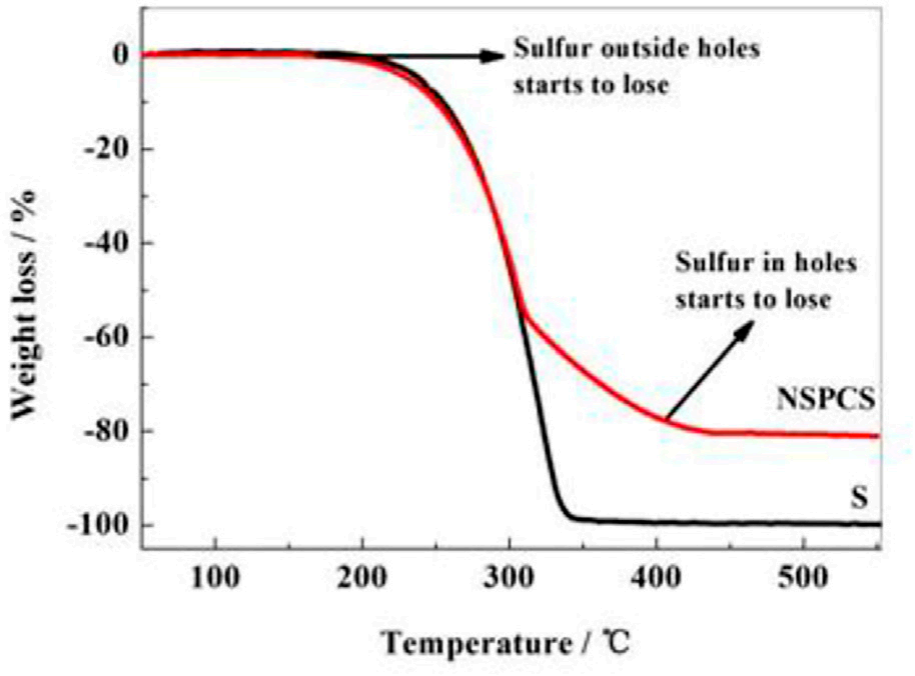
TG results of NSPCS and elemental sulfur.

The penetration of sulfur into the prepared NSPC matrix resulted from a hot melt-diffusion method. Thermogravimetric analysis was used to determine the sulfur content in the NSPCS sample as a percentage of total weight. The thermal weight loss period in Figure 5 was used to compute the sulfur content, which was discovered to be approximately 81%. Additionally, two weight loss platforms can be observed in the thermogravimetric curves between 160°C and 450°C, corresponding to the loss of sulfur from the outer layer and from the holes, respectively. Higher thermal stability of sulfur in NSPCS compared to that of elemental sulfur is due to the prevention of volatilization of sulfur by capillary force of the NSPC pores.

### Electrochemical performance

NSPCS composite and lithium metal was respectively used as positive and negative electrode of Li-S batteries to assemble into a 2032 coin cell. Electrochemical performance was conducted to study the influence of pore structure and heteroatom doping on the materials.

**FIGURE 6:**
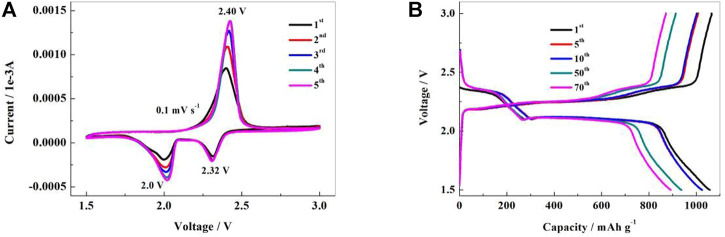
Cyclic voltammograms **(A)** and charge-discharge curves **(B)** of NSPCS electrode in the voltage range of 1.5–3 V (vs. Li/Li^+^).

In the first discharge, the CV curve of the NSPCS composite positive electrode material reveals reduction peaks at 2.0 V and 2.32 V which correspond to the transformation of elemental sulfur to high-valent polysulfide ions and further decrease to Li_2_S_2_/Li_2_S, respectively (Jayaprakash et al., 2011). For scanning in the opposite direction, the emergence of an oxidation peak near 2.40 V indicates the synthesis of polysulfide ions and their subsequent oxidation into elemental sulfur. Furthermore, the tight peak shape and overlapping of the first five CV curves indicated that the NSPCS positive electrode material had good electrochemical reversibility and reaction kinetics. These CV results indicate good electrical conductivity of the NSPCS composite which results in the inhibition of polysulfides dissolution during the charge-discharge process. Additionally, it can be seen that in the CV curve of Figure 6A, the current intensity increases as the reaction proceeds, which should be caused by insufficient electrolyte penetration.

Galvanostatic charge-discharge tests were further carried out to investigate the electrochemical behavior of the NSPCS composite. Figure 6B presents the charge-discharge curves of the NSPCS composite as positive electrode after 1st, 5th, 10th, 50th, and 70th cycle at discharge rate of 0.3 C (1 C = 1,675 mA·g^−1^). The first cycle discharge curve of the battery exhibits two clear discharge platforms that correlate to the reduction of elemental sulfur to Li_2_S and are in good agreement with the CV results presented in Figure 6A. For the first cycle at a discharge rate of 0.3 C, the NSPCS composite material demonstrated a discharge capacity of 1,058 mA·h·g^−1^, which is a respectable first cycle discharge capacity. The discharge curve shows two steady discharge platforms after 70 cycles, and the discharge capacity is still 892 mA·h·g^−1^, demonstrating the NSPCS composite positive electrode material’s excellent cycle stability.

**FIGURE 7:**
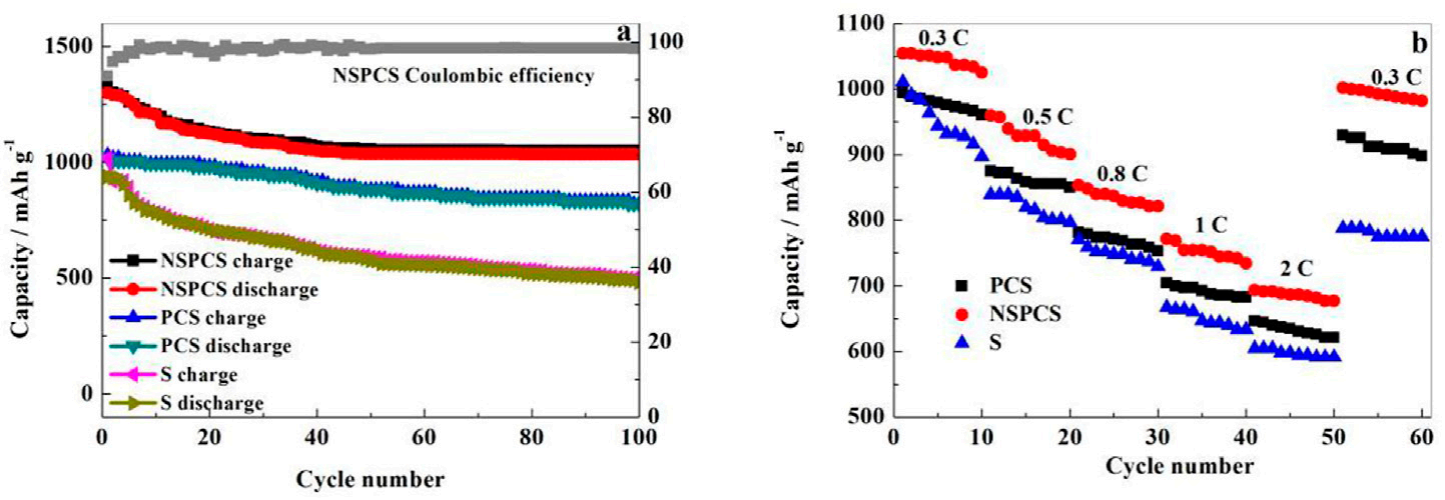
Cycling stability **(A)** and rate performances **(B)** curves of NSPCS, PCS and S electrode.

Figure 7A shows the performance of Li-S battery at 0.1 C over continuous number of cycles. As shown, the discharge capacity of NSPCS electrode is about 1,299 m·Ah·g^−1^ after 1^st^ cycle. The capacity after 100 cycles is retained at 1,052 mA·h g^−1^ with a capacity retention rate of around 81%. With respect to the Coulombic efficiency, the NSPCS composite materials showed good performance, retaining a value of 91% that of the first cycle. Figure 7B shows the rate performance of the discharge rates at room temperature for 0.3 C, 0.5 C, 0.8 C, 1 C, and 2 C. For the discharge rate of 0.3 C, NSPCS showed high discharge capacity of 1,054 mA·h·g^−1^ which decreased to 677 mA·h g^−1^ when the rate increased to 2 C. After continuously discharging at 0.5 C, 0.8 C, 1 C, and 2 C, when the rate of discharge was returned to 0.3 C again, the discharge capacity could still be restored to 980 mA·h·g^−1^. The rate performance is already very impressive. From the comparison result, it can be seen that whether the long cycle or rate performance, NSPCS is the best, followed by PCS, and both the two materials are better than sulfur electrode.

**FIGURE 8:**
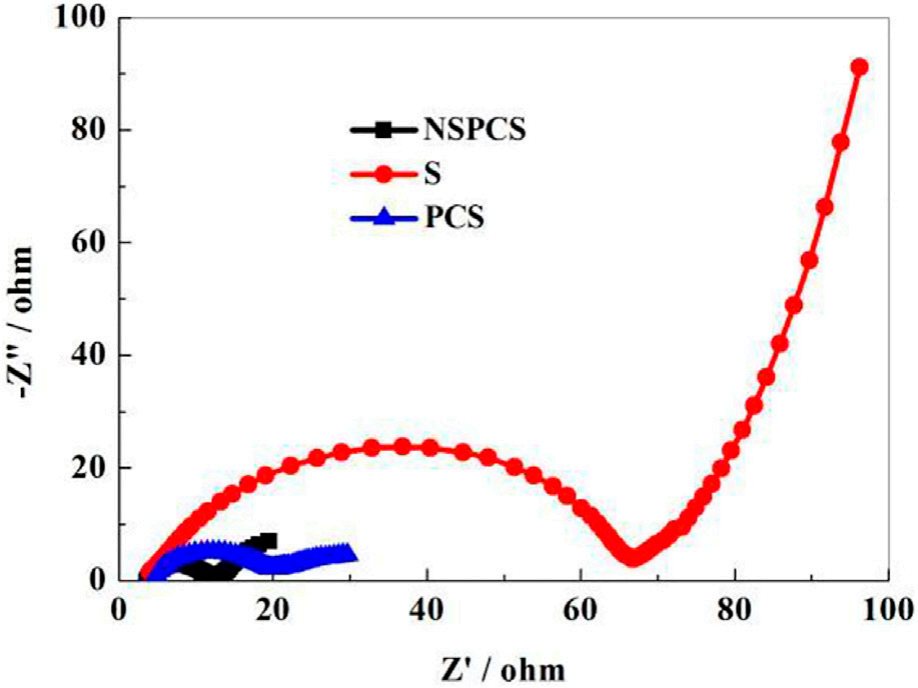
EIS results of NSPCS, PCS, and sulfur electrode.

The Figure 8 depicts the pre-discharge EIS spectra of NSPCS, PCS, and elemental sulfur electrodes (100 kHz–10 mHz). All the curves include two parts: a semicircle in the high frequency area and a straight line in the low frequency area. It can be seen that the ohmic impedance and charge transfer resistance of NSPCS and PCS both are smaller than that of sulfur electrode. The mixture of sulfur and the matrix improves conductivity significantly. Moreover, the resistance of NSPCS is smaller than PCS, indicating that the resistance of the material is further reduced when nitrogen and sulfur are doped.

## Conclusion

A porous carbon/sulfur doped with both nitrogen and sulfur (NSPCS) was synthesized *via* hydrothermal process in combination with KOH activation process by employing sucrose as carbon source, glutathione as nitrogen-sulfur source. Here, glutathione was not only the source of nitrogen and sulfur but also played the role of pore regulator to form carbon spheres with hierarchical microporous-mesoporous pore structure. More surface active sites can be introduced into carbon materials as a result of the addition of both nitrogen and sulfur, influencing microcrystalline conductivity, surface chemistry, and structure. The dissolution of polysulfide ions is greatly suppressed, along with the shuttle effects during the cycle of Li-S batteries, as a result of the strong interaction between sulfur and polysulfide ions at the interface of nitrogen-sulfur dual doped carbon material, improving cycle stability. The experimental results show that the synthesized N, S dual-doped porous carbon/sulfur (NSPCS) composite material has a regular spherical morphology, a large specific surface area, a hierarchical pore structure, and a high sulfur utilization rate, resulting in outstanding electrochemical performance.

## Data Availability

The original contributions presented in the study are included in the article/supplementary material, further inquiries can be directed to the corresponding authors.
